# The Blood-Count

**Published:** 1901-06-29

**Authors:** 


### THE BLOOD-COUNT.

The practical man is apt to scorn science and trust to what he chooses to call experience. It is well, however, to be reminded now and again of the important aids which may be gained from the judicious application of scientific methods of diagnosis. In his oration on surgery, delivered before the annual meeting of the American Medical Association, Dr. John A. Wyeth applied himself to demonstrate the value of clinical microscopy, bacteriology, and chemistry in surgical practice. Speaking of the methods at our disposal, for example, in forming a diagnosis between typhoid fever and appendicitis in some cases, he pointed out how often the safety of a patient hung upon even a few hours' time, and how often this precious time was wasted in the uncertainties of diagnosis while a resort to the demonstration of science would have plainly indicated the proper method of procedure. The ordinarily recognised symptoms may afford us no accurate clue to the pathological process which may exist. The pulse and temperature of commencing typhoid may well be mistaken for the pulse and temperature of an appendicitis. The pain and muscular resistance over the right iliac and the right abdominal region are in many instances practically alike in both cases. The nausea, vomiting, and the sense of uneasiness point neither directly to the one or the other, but in a crucial test by Widal's reaction, with the blood-count pointing to the presence or absence of a leucocytosis, the question is soon settled. "I have seen," he says, "all the symptoms of appendicitis present in cases in which the blood-count contradicted a pyogenic sepsis, and in which Widal's reaction told the story of typhoid. On the contrary I have dealt with cases which ordinarily would have been most perplexing in which all the symptoms of typhoid prevailed at a period when it was too early to recognise this disease by Widal's test, and a leucocytosis of from 15,000 to 21,000 proved at the earliest possible moment that the case was one for immediate operation." Referring more particularly to the importance of the blood-count, he goes on to say that in the early recognition of the septic processes—chiefly pyogenic—surgery can no longer disregard the value of the blood-count, especially the estimation of the leucocytes. After hæmorrhage the leucocyte count is increased, and in diphtheria, erysipelas, trichinosis, all extensive forms of endometritis, and all acute pyogenic processes, leucocytosis exists, except in those cases where the vitality of the individual has been overwhelmed by the severity of the septic process, under which condition the leucocytes no longer respond to the demand for the protection of the tissues and are not present in the superficial blood even in normal proportions. In no cases are these facts capable of a more practical application than in the diagnosis of diseases of the abdominal and thoracic organs. In a certain proportion of cases of infection temperature does not always indicate the increasing gravity of the lesion, while the degree of sepsis can be in great measure determined by the leucocyte count. In impaction of fæces, extra-uterine pregnancy, floating kidney, gall-stone, renal colic, ovarian neuralgia, intussusception, volvulus, inguinal hernia, twisted pedicle, etc., there is no leucocytosis, unless complicated with an acute septic process.

